# The effect of coconut oil and palm oil on anthropometric parameters: a clinical trial

**DOI:** 10.1186/s40795-023-00812-y

**Published:** 2024-01-10

**Authors:** Hasinthi Swarnamali, Priyanga Ranasinghe, Ranil Jayawardena

**Affiliations:** 1 Health and Wellness Unit, Faculty of Medicine, University of Colombo, No 25, Kynsey Road, Colombo 08, Colombo, Sri Lanka; 2 Department of Pharmacology, Faculty of Medicine, University of Colombo, Colombo, Sri Lanka; 3 University/British Heart Foundation Centre for Cardiovascular Science, The University of Edinburgh, Edinburgh, UK; 4 Department of Physiology, Faculty of Medicine, University of Colombo, Colombo, Sri Lanka; 5 Institute of Health and Biomedical Innovation, Queensland University of Technology, Brisbane, QLD Australia

**Keywords:** Coconut oil, Palm oil, Clinical trial, Anthropometric parameters, Bodyweight

## Abstract

**Background:**

During recent years several studies have investigated the impact of different dietary oils on body weight. They have shown differential positive and negative effects on anthropometry. We investigated the effects of palm and coconut oils on body weight and other anthropometric parameters, considering their importance as a primary source of saturated fat, controlling for other confounding variable such as total energy intake.

**Methods:**

The study was conducted as a sequential feeding clinical trial with 40 healthy men and women divided into two feeding periods of initial palm oil (8 weeks) and subsequent coconut oil (8 weeks), with a 16-week washout period in between. Each participant received a pre-determined volume of each oil, which were integrated into their routine main meals and snacks during the respective study periods. Changes in body weight, body mass index (BMI), waist circumference (WC), hip circumference (HC), and waist-to-hip ratio (WHR) were evaluated. Physical activity levels and dietary intake were also evaluated as potential confounding factors.

**Results:**

Thirty-seven participants completed both oil treatment periods. The mean (± SD) age of the participants was 39 (± 13.1) years. There were no significant differences in any of the anthropometric parameters between the initial point of feeding coconut oil and the initial point of feeding palm oil. Following both oil treatment phases, no significant changes in the subjects’ body weight, BMI, or other anthropometric measurements (WC, HC, and WHR) were observed.

**Conclusion:**

Neither coconut oil nor palm oil significantly changed anthropometry-related cardiovascular risk factors such as body weight, BMI, WC, HC, and WHR.

**Trial registration:**

Sri Lankan Clinical Trial Registry: SLCTR/2019/034 on 4th October 2019 (https://slctr.lk/trials/slctr-2019-034).

**Supplementary Information:**

The online version contains supplementary material available at 10.1186/s40795-023-00812-y.

## Introduction

Several recent studies have examined the impact of different dietary oils on body weight, including virgin coconut oil [1], olive oil [2], canola oil [3], and palm oil [4], considering the varying constituents present in these oils that are associated with adiposity. For instance, several studies have reported an inverse association between omega-3 polyunsaturated fatty acids (PUFAs) and weight gain [5–7] due to its effects on fat oxidation [8] and postprandial satiety in the overweight and obese people [9]. However, further to the above postulated positive effect of oil constituents on body weight, certain fatty acid constituents are known to have a negative impact. For example, the consumption of saturated fat has been consistently linked with the development of obesity [10]. Furthermore, long chain saturated fatty acids like myristic, palmitic, and lauric acid found in both palm and coconut oils have been linked with weight gain when compared with short-chain saturated fatty acids and unsaturated fatty acids like oleic acid [11]. Therefore, due to the high content of saturated fatty acids, it can be postulated that palm oil and coconut oil, when consumed regularly increases the risk of obesity [12].

Coconut and palm oils are the primary sources of dietary saturated fatty acids (SFA) among South-East Asians [13]. People from Sri Lanka, India, Indonesia, and the Philippines use these two oils on a regular basis for culinary purposes. In addition they are used during the manufacturing of processed food such as margarine, bakery items, and confectionaries. Although both oils are high in SFAs, there are differences in the fatty acids that make up each oil’s composition. Coconut oil is composed of around 90% saturated fats [14]. The medium-chain fatty acid (MCFA) lauric acid (C12:0) is the fatty acid with the highest concentration, accounting for 49% of all fatty acids found in coconut oil [15]. In contrast, palm oil contains 10% PUFAs, 40% monounsaturated fatty acids, and 50% SFAs [11]. Therefore, regular palm oil, has an approximately balanced proportion of unsaturated fats (USFAs) (50%) and SFAs (50%), with 44% palmitic acid (C16:0), 5% stearic acid (C18:0), and traces of myristic acid (C14:0) as SFAs, and it is high in antioxidants, beta-carotene, and vitamin E [11]. Palmitic acid the primary fatty acid in palm oil is classified as an LCFA, whereas lauric acid the most common fatty acid in coconut oil is categorised as an MCFA due to their biochemical characteristics [16]. However, when digestion and absorption is considered, lauric acid is also classified as an LCFA, since 70–75% of it is absorbed via chylomicrons [17]. Furthermore, MCFAs are less efficient in promoting fat storage and more vulnerable to oxidative metabolism than LCFAs [16]. Therefore, it can be postulated that MCFAs less likely to result in substantial weight gain due to adipocyte accretion [16].

Although based on the biochemical classification coconut oil can be considered as a thermogenic food that helps burn more calories, [18], a recent meta-analysis carried out to determine the effect of coconut oil on anthropometric parameters in comparison to other edible oils found that only few anthropometric parameters, such as body weight, body mass index, and fat mass percentage, had a statistically significant reduction with coconut oil, while no statistically significant change was observed with waist circumference, waist to hip ratio, or fat mass [19]. Furthermore, because palm oil also includes a high percentage of saturated fat, it is thought to raise blood cholesterol levels, increasing the risk of cardiovascular disease [20]. However, our ability to draw conclusions about the role of palm oil on obesity is currently constrained, since there are no clinical studies that compare the association between body weight and palm oil intake. Comparing the effects of palm oil and/or coconut oil on body weight and other anthropometric measurements is important because they are major sources of saturated fat in many developing countries. Therefore, the objective of the current study was to assess and compare the effects of coconut oil and palm oil in a sequential feeding clinical trial, elucidating their effects on body weight and other measurements. We hypothesize that there will be significant differences in anthropometric measurements, such as body weight, body mass index (BMI), waist circumference (WC), hip circumference (HC), and waist to hip ratio (WHR), between individuals consuming coconut oil or palm oil.

### Methodology

Ethics approval for the study was granted by the Ethics Review Committee, Faculty of Medicine at the University of Colombo (EC-19-046) and the clinical trial in the Sri Lanka Clinical Trials Registry (SLCTR/ 2019/ 034) [21]. The detailed methodology has been published elsewhere [22].

#### Study setting and participants

This experimental study was conducted in the Galle district, Southern province of Sri Lanka from February to August 2021. Forty healthy adults (> 18 years of age, both male and female) from the community were invited for the study. Demography, medical history and co-morbidities, dietary habits, and usual physical activity patterns of each subject were collected using a standard interviewer-administered questionnaire. Those who satisfied the initial eligibility criteria (Supplementary Table 1) were invited to a health screening session. Informed written consent was obtained from all subjects prior to recruitment.

#### Sample size

The sample size for this study was calculated based on the primary outcome measure of change in mean low density lipoprotein cholesterol (LDL-C) level. While this paper emphasizes anthropometric measurements, the comprehensive study also includes an evaluation of plasma lipoproteins and other biochemical parameters, which will be detailed in a separate publication. The mean LDL-C (primary outcome measure) change in the coconut oil feeding group was 10 mg/dL while it was − 15 mg/dL in the palm oil group in the reference study [23]. Therefore, 25 mg/dL was the expected difference in LDL-C between coconut oil and palm oil feeding groups (P < 0.001) as per the reference study [23] and this was considered for sample size estimation for this trial. According to the statistical calculation of the sample size to detect a 25 mg/dL in serum LDL-C difference between the two feeding periods of coconut and palm oil, while maintaining the type one error (α) at 0.05 and type 2 error (β) at 0.1 and thereby having a power of 90%, the required number of subjects in the trial was 32. We assumed a dropout rate of 20% and therefore 40 subjects were recruited for this study.

#### Intervention

The intervention period was structured into two 8-week feeding phases. During the first feeding period, participants received palm oil. Following the feeding period for palm oil, there was a 16-week washout period. Participants were allowed to use their usual oil during this period. After that, coconut oil was used for another 8 weeks. To assess the daily amount of oil used during food preparation, a typical macronutrient profile among Sri Lankans was considered [24] and therefore, 19% of daily energy was considered from the total fat in the diet, and from that 50% was considered as being taken from treatment the oil. Therefore, nearly 10% of total daily energy was considered as being from the test oils. Based on the participant’s average calorie intake as determined by a 24-hour dietary recall, the minimal amount or dosage of the oil required was calculated. We did not give any prescribed diet as we evaluated changes in a ‘real world’ situation among free-living individuals. The diets consisted of foods containing palm oil for the main meals and snacks during the first interventional period while coconut oil was used in the second interventional period. Adherence to the intervention plan was checked by using food recalls, interviews, and pictures of the oil recording sheet and oil bottle (with remaining oil) at the end of each week. Copra coconut oil and palm olein were used for the current study. Both coconut and palm oil used in the study were taken from a single batch, and the fatty acid composition in both oil samples were analysed (Supplementary Table 2).

### Outcome assessment

The detailed items that were assessed during each visit are outlined in Supplementary Table 3. Details of the Socio-demographic details and anthropometric assessment are presented in Supplementary Content 1. A culturally validated food frequency questionnaire (FFQ) [25] and the 24 h dietary recalls were applied for dietary data and subsequently analysed using open-access Nutri-Survey 2007 nutrient analysis software (EBISpro, Willstaett, Germany) [25]. The validated IPAQ (International Physical Activity Questionnaire-long version) was used to assess the level of physical activity [26].

### Data analysis

The statistical analysis was conducted using SPSS version 22 (SPSS Inc., Chicago, IL, USA). Analytical results were determined and are presented as mean (± SD), with a p-value < 0.05 being considered as statistically significant. The change in the means (delta value) of the specific treatments on weight, BMI, WC, HC, and WHR were compared using a paired sample t-test to assess the impact of two treatments over the course of the intervention. Data from the IPAQ, FFQ, and 24-hour dietary recall during the two different treatments were also compared by paired sample *t*-test. All statistical analysis was as per protocol and included only those volunteers who had completed both phases without major protocol deviations and with more than 80% compliance throughout the intervention. Therefore, volunteers with any missing values for these parameters were removed.

#### Monitoring the compliance of the oil intake

Subjects were asked to maintain daily records of oil volume in an oil record sheets. Oil records also were checked using photos weekly. Oil compliance for the number of days with treatment oil and the percentage of daily energy from test oil were evaluated. Detailed information on monitoring the compliance and calculation of compliance for the following are presented in Supplementary Content 2. The oil compliance was evaluated by two methods;

1. Compliance for the number of days with treatment oil and.
2. Compliance with the percentage of daily energy from test oil.

The compliance rate of more than 80% for both parameters were considered in the final analysis.

## Results

**Fig. 1:**
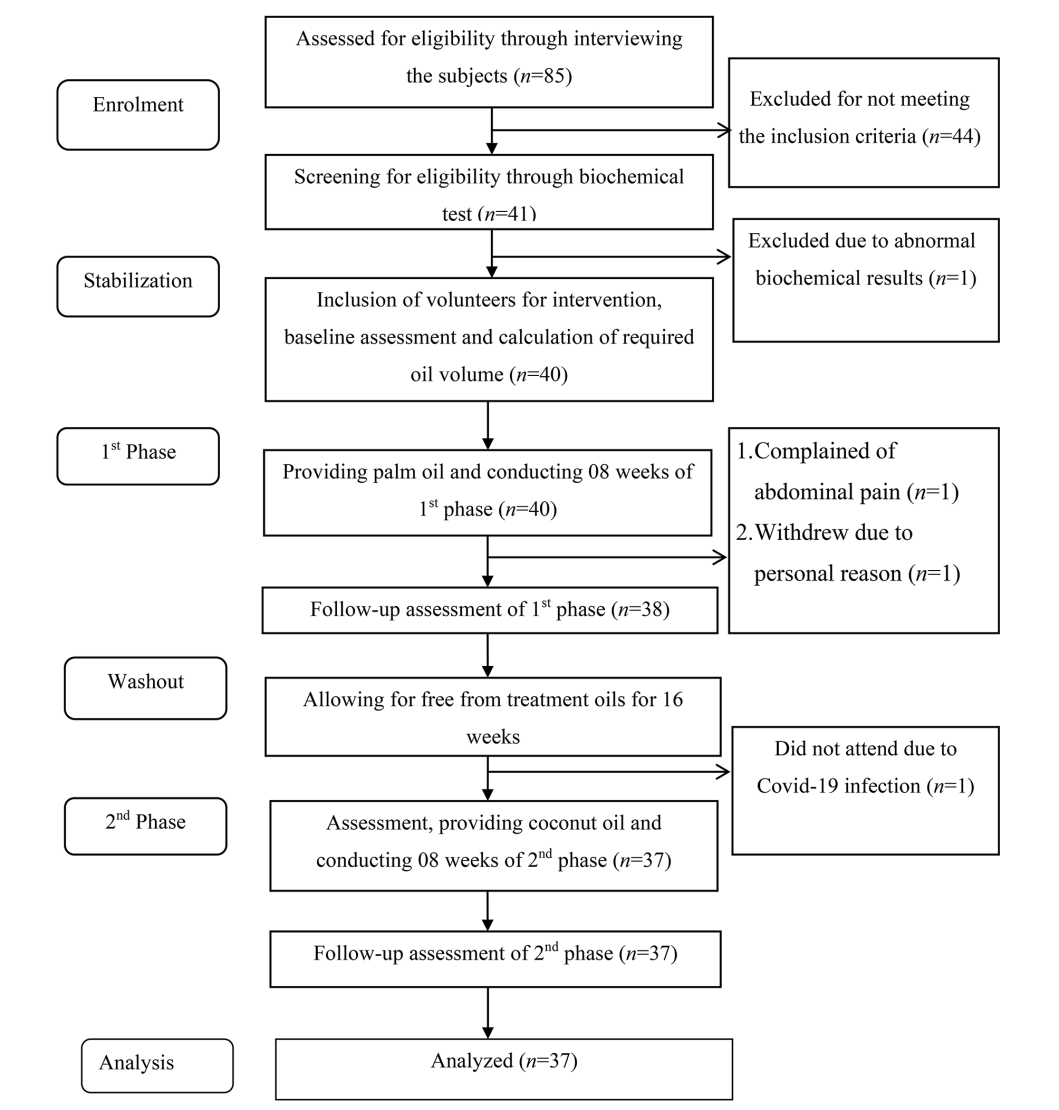
Recruitment and flow diagram of the clinical trial with palm oil and coconut oil

Participant enrolment and follow-up details are summarized in Fig. 1. The baseline information of the study participants are presented in Table 1. Of the sample 81.1% (*n* = 30) were married, 62.2% (*n* = 23) were females, and 64.9% (*n* = 24) had tertiary level education, while 43.2% (*n* = 16) stated that they were self-employed. The majority of the population (54.1%, *n* = 20) were normal weight according to Asian BMI cut-off values. The mean (± SD) age of the participant were 39 (± 13.1) years, while mean weight, height and BMI were 59.5 (± 11.7) kg, 158.2 (± 9.6) cm, BMI was 23.8 (± 4.6) kg.m^− 2^ respectively at baseline. Socio-demographic characteristics and family history of co-morbidities of the participants are summarised in Supplementary Table 4.

**Table 1:** Baseline characteristics of the study population (n = 37)

Variable	Mean (± SD)*	Range
Age (years)	39 (± 13.1)	19 − 72
Weight (kg)	59.5 (± 11.7)	37.9–78.0
Height (cm)	158.2 (± 9.6)	138.2–179.9
Body Mass Index (kgm^− 2^)	23.8 (± 4.6)	16.3–35.8
Waist circumference (cm)	86.6 (± 12.7)	58.8–111.0
Hip circumference (cm)	92.1 (± 8.7)	75.5– 113.0
Waist to hip ratio	0.94 (± 0.09)	0.75–1.11

* Mean (± standard deviation) of 37 participants

### Comparison of anthropometric parameters between treatments

Table 2 summarizes the data on anthropometric measurements at four time points, namely the beginning and end of each oil treatments. There were not significant variations in any of the anthropometric parameters from the initial measurements for both coconut oil and palm oil. Body weight, BMI, and other anthropometric measurements (WC, HC, and WHR) did not change significantly following either oil treatment period. Table 3 compares and summarizes the outcomes of each treatment oil on anthropometric parameters.

**Table 2:** Impact of coconut oil and palm oil on anthropometric measures

Anthropometric variable		Mean (± SD)			Mean change (T_2_– T_1_)	Δ %^a^	*p* value^b^
		Initial (T_1_)		Final (T_2_)			
Weight (kg)	Palm oil (*n* = 37)	59.55 (± 11.68)	59.34 (± 11.54)		-0.21 (± 0.93)	-0.35%	0.183
	Coconut oil (*n* = 37)	59.29 (± 11.48)	59.34 (± 11.40)		0.04 (± 1.07)	+ 0.08%	0.820
	*p* value^c^	0.372	0.992		0.331		
Body mass index (kgm^− 2^)	Palm oil (*n* = 37)	23.84 (± 4.59)	23.76 (± 4.56)		-0.08 (± 0.38)	-0.34%	0.228
	Coconut oil (*n* = 37)	23.76 (± 4.65)	23.78 (± 4.61)		0.02 (± 0.44)	+ 0.08%	0.828
	*p* value^c^	0.538	0.871		0.376		
Waist circumference (cm)	Palm oil (*n* = 37)	86.58 (± 12.66)	87.02 (± 12.35)		0.44 (± 3.00)	+ 0.51%	0.384
	Coconut oil (*n* = 37)	87.51 (± 11.87)	86.95 (± 11.71)		-0.57 (± 2.85)	-0.64%	0.234
	*p*value^c^	0.171	0.881		0.19		
Hip circumference (cm)	Palm oil (*n* = 37)	92.09 (± 8.69)	91.82 (± 8.11)		-0.27 (± 2.07)	-0.29%	0.437
	Coconut oil (*n* = 37)	93.77 (± 8.56)	93.95 (± 8.39)		0.18 (± 1.64)	+ 0.19%	0.520
	*p* value^c^	< 0.001	< 0.001		0.365		
Waist to hip ratio	Palm oil (*n* = 37)	0.95 (± 0.09)	0.94 (± 0.08)		0.01 (± 0.04)	-1.05%	0.270
	Coconut oil (*n* = 37)	0.93 (± 0.07)	0.92 (± 0.07)		-0.01 (± 0.03)	-1.07%	0.100
	*p* value^c^	0.331	< 0.001		0.07		

^a^Δ % = [(T_2_ -T_1_) /T_1_] x 100 or relative mean change between final and initial data, “-” represent decrease and “+” represents an increase

^b^*p*-value is for final vs.initial comparison. Data were analyzed by paired sample *t*-test and *p* < 0.05 was considered significant

**Table 3:** Summary of comparison between treatments

Anthropometric parameters	Mean (± SD) difference (Final - Initial)		*p* value
	Palm oil	Coconut oil	
Weight (kg)	-0.21 (± 0.93)	0.04 (± 1.07)	0.331
BMI (kg/m^2^)	-0.08 (± 0.38)	0.02 (± 0.44)	0.376
Waist circumference (cm)	0.44 (± 3.00)	-0.57 (± 2.85)	0.19
Hip circumference (cm)	-0.27 (± 2.07)	0.18 (± 1.64)	0.365
Waist to hip ratio	0.01 (± 0.04)	-0.01 (± 0.03)	0.07

Mean (± SD) differences (*n* = 37) between final and initial measurements (Delta value) of the respective treatment groups (i.e. palm oil and coconut oil) were subjected to paired sample *t-*test (*p* < 0.05). *Abbreviation*: SD–standard deviation

### Nutrient intake and physical activity level

Food intake and level of physical activity were measured as potential confounding variables to compare them between two treatment periods (Table 4 and Table 5).

**Table 4:** Dietary profiles of participants before and after feeding the treatment oils during 8 weeks using 24-hour dietary recall

Confounding variables	24-hour dietary recall	Study population		*p* value^b^
		Palm oil (*n* = 37)	Coconut oil (*n* = 37)	
Total energy (kcal/day)	Initial evaluation (T_1_)^a^	1628.7 (± 214.7)	1707.9 (± 187.7)	0.055
	Final evaluation (T_2_)^a^	1601.4 (± 128.9)	1744.3 (± 295.8)	< 0.001
	*p* value^b^ (Δ %^c^)	0.414 (-1.7% )	0.004 (+ 2.1%)	
Carbohydrate (g/day)	Initial evaluation (T_1_)^a^	282.7 (± 21.6)	289.1 (± 16.1)	0.169
	Final evaluation (T_2_)^a^	280.1 (± 13.6)	288.4 (24 ± 0.4)	0.112
	*p* value^b^ (Δ %^c^)	0.510 (-0.9%)	0.839 (-0.2%)	
Protein (g/day)	Initial evaluation (T_1_)^a^	47.1 (± 10.6)	53.0 (± 12.4)	0.012
	Final evaluation (T_2_)^a^	48.2 (± 12.9)	54.6 (± 12.3)	0.016
	*p* value^b^ (Δ %^c^)	0.658 (+ 2.3%)	0.011 (+ 3.1%)	
Fat (g/day)	Initial evaluation (T_1_)^a^	33.9 (± 9.4)	37.7 (± 13.5)	0.124
	Final evaluation (T_2_)^a^	34.1 (± 5.4)	41.2 (± 6.1)	< 0.001
	*p* value^b^ (Δ %^c^)	0.098 (+ 0.6%)	0.043 (+ 9.1%)	
Dietary fibre (g/day)	Initial evaluation (T_1_)^a^	14.1 (± 6.2)	15.0 (± 4.8)	0.335
	Final evaluation (T_2_)^a^	12.8 (± 6.4)	15.6 (± 5.1)	0.008
	*p* value^b^ (Δ %^c^)	0.226 (-8.9%)	0.099 (+ 4.2%)	

^a^Mean values (± standard deviation) of 37 subjects

^b^According to one-way repeated measures ANOVA (p < 0.05)

^c^Δ % = [(T_2_ - T_1_) /T_1_] x 100 or relative mean difference between after and before the intervention, “-” represent decrease, “+” represents an increase

**Table 5:** Dietary intake and physical activity level (confounding variables) during coconut and palm oil-periods

Confounding variable		Mean (± SD)^a^		*p* value^b^
		Palm oil (*n* = 37)	Coconut oil (*n* = 37)	
Dietary intake*	Total energy (kcal/day)	1603.45 (± 438.4)	1606.42 (± 435.3)	0.280
	Carbohydrate (g/day)	282.08 (± 80.0)	283.16 (± 78.8)	0.103
	As a percentage from TE (%)	70.1 (± 5.5)%	69.4 (± 5.1)%	
	Protein (g/day)	47.79 (± 19.2)	48.22 (± 18.8)	0.173
	As a percentage from TE (%)	11.3 (± 3.0)%	11.9 (± 5.1)%	
	Fat (g/day)	32.27 (± 11.9)	32.81 (± 11.7)	0.160
	As a percentage from TE (%)	18.4 (± 4.4)%	18.2 (± 4.0)%	
	Dietary fibre (g/day)	16.75 (± 6.3)	20.79 (± 7.3)	< 0.001
Physical activity (MET-minutes/week)**		3943.74 (± 3651.0)	3574.46 (± 3762.4)	0.082

^a^ Mean (± SD)

^b^According to the paired sample *t*-test (*p* < 0.05)

MET minutes/week- Metabolic Equivalent-Minutes per week

*Dietary intake was measured using the Food Frequency Questionnaire (FFQ)

**Physical activity was measured using International Physical Activity Questionnaire (IPAQ)

### Mean oil intake and oil compliance

The mean (± SD) percentage of energy intake from oils and percentage energy intake from oils with reference to the FFQ is presented in Table 6. The oil compliance rate (%) of the population was evaluated using oil recordings in terms of the number of days of oil consumed and the percentage of energy intake from test oil (Table 7).

**Table 6:** Mean energy percentage from test oils compared to total energy intake based on 24-hour dietary recall and Food Frequency Questionnaire

	Mean (± SD), %	
	24-hour dietary recall	FFQ
Palm oil	12.5 (± 2.0)	12.9 (± 4.1)
Coconut oil	12.2 (± 2.5)	13.3 (± 4.0)
*p*-value	0.550	0.331

Abbreviation: FFQ- food frequency questionnaire; SD- standard deviation

**Table 7:** Compliance of test oil intake

	Mean (± SD), %		
	Number of oil intake days as (%)^a^	Energy from test oil (%)^b^	
		24-hour dietary recall	FFQ
Palm oil	97.7 (± 2.9)	99.9 (± 0.1)	95.5 (± 9.3)
Coconut oil	98.4 (± 2.5)	98.6 (± 4.0)	96.6 (± 9.6)
*p* value	0.079	0.038	0.157

Percentage (%) of mean compliance was subjected to all 37 subjects and *p*-value was based on paired sample *t*-test

^a^Compliance % (as days consumed of test oil) = [(no. of days with treatment oil– no. of days without treatment oil)/ No of days with treatment oil] x 100%

^b^Compliance % (as energy % from test oil) = [Average daily energy intake from test oil (%)* / Minimum percentage of recommended energy from test oil ( or 10%)] x 100%

*Average percentage of daily energy intake from test oil (%) = [Average daily energy intake from test oil (kcal)^#^ /Average total daily energy intake from the complete diet (kcal)] x100%

^#^Average daily energy intake from test oil (kcal) = {Total volume of oil consumed for treatment period (mL)/ [(no. of days x number of members consumed per day)]} x 0.92 × 9 kcal/g

Abbreviation: FFQ- food frequency questionnaire; SD- standard deviation

## Discussion

In the present sequential feeding trial, adults from the general community were assigned to one of two cooking oils each for 8 weeks, interspersed with a 16-week wash-out phase to compare the effect of the individual oils on anthropometric variables. To the best of our knowledge, there are no previous human clinical trials comparing the effects of palm and coconut oils on changes in anthropometric outcomes in humans. However, a single animal study that (male Wistar rats) has also reported that there were no statistically significant variations in anthropometric data such as BMI, WC, and fat mass when palm and coconut oil were compared [27].

The chemical composition of palm and coconut oil, notably the chain length and saturation level of fatty acids, is postulated to contribute towards their impact on anthropometric parameters [28]. Fatty acids, classified as SFA, MUFA, and PUFA, exhibit distinct effects on cholesterol levels [29]. Further categorization based on carbon length divides them into short (C2–C6), medium (C8-C12), and long (C14–C24) chain fatty acids [30]. MCFA, with 7–12 carbons, are efficiently absorbed and rapidly oxidized in the liver, promoting potential weight control [31]. In contrast, LCFAs rely on the carnitine shuttle for mitochondrial delivery, resulting in adipose tissue absorption [32]. Substituting MCFAs for LCFAs in the diet can influence metabolic pathways, inducing satiety and increasing energy expenditure [32].

With regard to chain length, both coconut and palm oils are simple and economical sources of MCFAs among other plant oils [33]. Various sources attribute coconut oil’s therapeutic effects to its greater concentration of MCFAs, which account for 64% of overall fats [34]. Coconut oil had the greatest concentration of MCFAs in a comparison study of five cooking oils, including sunflower, soybean, palm, mustard, and coconut oils [35]. In addition, a comprehensive examination of fourteen different cooking oils revealed that coconut oil had the highest proportion of MCFAs and the lowest amount of LCFAs [36]. However, the current study did not show a significant beneficial outcome of coconut over palm oil on anthropometric measurements.

The dietary profiles of participants, assessed through 24-hour dietary recalls and FFQs at various time points, showed a consistent total energy intake and macronutrient consumption between palm oil and coconut oil feeding periods. This uniformity suggests stable calorie and macronutrient intake, minimizing confounding effects on anthropometric variables. Physical activity levels, assessed via IPAQ, exhibited no significant variation during palm and coconut oil-treated periods, ruling out confounding influences on anthropometric parameters in this study. The mean energy percentage from test oils, calculated through 24-hour dietary recall and FFQ, consistently met the recommended dosage of at least 10% of total energy. The oil dosage and compliance were similar during both treatment periods. Compliance, assessed for both energy intake and the number of days consumed, exceeded 95% for the study population.

Several strengths of the present study need to be highlighted. We observed a high compliance rate to the test oils throughout both interventional periods. This was a community-based interventional study in a normal free-living population with both genders, with a reasonable age distribution, including both young and old. In contrast to previous clinical trials comparing the effect of coconut and palm olein oils on cardio-metabolic health effects where participants were predominately young [37–40], only females [39], and non-matched genders in others [37–40]. Several studies have shown that serum lipid levels are influenced by age and gender, therefore, in such studies, it is important to include a representative sample inclusive of both genders with an appropriate age distribution [41, 42]. Neither the level of physical activity nor nutrient consumption significantly differed, minimizing their potential confounding influence on study outcomes. The trial, conducted on volunteers from the general community, might introduce volunteer bias, but this bias is mitigated by the likelihood of better adherence to the trial protocol and investigator advice. This paper finds that the consumption of widely used coconut oil and palm oil does not significantly affect key anthropometric measurements. However, for a comprehensive understanding of cardiovascular health, it is important to consider additional data on plasma lipoproteins, which is addressed in companion papers from the same study.

### Limitations

However, few limitations need to be acknowledged. Firstly there is limited data quantifying fat content from various sources in Sri Lankan diets, therefore, an assumption was made that other fat-containing foods contributed 50% of energy from total fat sources. The remaining 50% of energy was supplied through the test oil, calculated considering 19% of total daily energy from fat based on available study [24]. Acknowledging the lack of national-level data, this approach provides an approximate calculation. However, it’s important to note that assuming a 50% energy contribution from the test oil may not be accurate due to daily variations in individual food consumption. Additionally, the Nutri Survey Software used for dietary analysis was not validated for dietary fat composition, lacking specific details on PUFA, MUFA, SFA, and trans fat levels at the time of data analysis, given the absence of a validated Sri Lankan food/nutrient analysis database/tool for accurate interpretation of dietary fat composition.

## Conclusions

In conclusion, our study aimed to investigate the effects of coconut oil compared to palm oil on anthropometric-related cardiovascular risk factors, including body weight, BMI, WC, HC, and WHR. The results revealed that neither coconut oil nor palm oil had any significant effect on these parameters. Moreover, the observed non-significant changes in dietary intake and physical activity levels provide assurance against potential confounding variables influencing study outcomes. These collective findings contribute valuable insights and set a foundation for future clinical trials exploring the nuanced effects of different oils on cardiovascular health-related anthropometric measures.

### Electronic supplementary material

Below is the link to the electronic supplementary material.

Supplementary Material 1

## Data Availability

Not applicable. The raw de-identified data may be made available upon reasonable request from the corresponding author.

## Abbreviations

SD: Standard deviation
TE: Total energy
