# Supplementary material for: The effect of coconut oil and palm oil on anthropometric parameters: a clinical trial
Source: BMC Nutr. 2024 Jan 10;10:9. doi: 10.1186/s40795-023-00812-y (PMC10782760; doi:10.1186/s40795-023-00812-y)
Supplement: Supplementary file 1 — Supplementary Material 1 [file 40795_2023_812_MOESM1_ESM.docx]

Supplementary Table 1: Inclusion and exclusion criteria

|  | **Exclusion criteria** | **Inclusion criteria** |
| --- | --- | --- |
| **1** | A history of atherosclerotic CVD, endocrine diseases including thyroid disease and type 1 or 2 diabetes mellitus, | Healthy adults (>18 years) in free-living general community. |
| **2** | Uncontrolled hypertension | Those who were not having a history of allergy to coconut oil and palm oil. |
| **3** | An abnormal laboratory test result of clinical significance | Those who were willing to consume both coconut and palm oils in a sequential pattern follow the dietary instructions (as per protocol). |
| **4** | A history/presence of any clinically significant pulmonary disease, chronic inflammatory, hepatic, renal, hematologic, immunologic, dermatologic, neurologic, psychiatric, malignancy or biliary disorder | Those who did not restrict oil for their usual cooking practices agreed not to restrict oily foods during the study period. |
| **5** | Individuals who were taking any medications for dyslipidemia (as that has a direct effect on the outcome measure), medications known to influence glucose metabolism, systemic corticosteroids, antihypertensive medicines, or nonsteroidal anti-inflammatory drugs | Those who took all three main meals at home. |
| **6** | Those who used foods or herbal or dietary supplements (such as antioxidant or vitamin or mineral or fish oil or omega 3 or omega 6 supplements) that might alter lipid metabolism | Those who were willing to maintain their usual dietary intake, habits, and physical activity level throughout the intervention period |
| **7** | Those who used weight-loss medications or followed life-style programs for weight loss | Those who don’t use alcohol or tobacco. |
| **8** | Those who followed a prescribed diet |  |
| **9** | Those who frequently consumed non-home cooked foods (at least 2 days per week) |  |
| **10** | Female subjects who were pregnant, lactating, and planning to be pregnant during the study period |  |
| **11** | Individuals who had extreme dietary habits (severely reduced calorie intake or main meals restricted to juice or single food or green leafy or carbohydrate-free diet) |  |
| **12** | Those who had been diagnosed with eating disorders |  |

Supplementary Table 2: Fatty acid profile of oils used in the study

| Major fatty acids | Percent by mass (g/ 100 g of fat) | |  |
| --- | --- | --- | --- |
|  | Palm oil | Coconut oil |  |
| Caproic acid (C6:0) | ND | 0.45 |  |
| Caprylic acid (C8:0) | ND | 8.22 |  |
| Capric acid (C10:0) | ND | 5.71 |  |
| Lauric acid (C12:0) | 0.31 | 49.10 |  |
| Myristic acid (C14:0) | 1.02 | 18.26 |  |
| Palmitic acid (C16:0) | 36.56 | 8.13 |  |
| Palmitoleic acid (C16:1) | 0.22 | ND |  |
| Stearic acid (C18:0) | 3.75 | 2.59 |  |
| Oleic acid (C18:1c) | 43.98 | 5.99 |  |
| Elaidic (C18:2 (9c,12t) | 0.11 | ND |  |
| Linoleic (C18:2 (9t,12c) | 0.10 | ND |  |
| Linolelaidic (C18:2 n6c) | 12.90 | 1.55 |  |
| Linolenic (C18:3) | 0.60 | ND |  |
| Arachidic acid (C20:0) | 0.32 | ND |  |
| Behenic acid (C22:0) | 0.06 | ND |  |
| Lignoceric acid (C24:0) | 0.07 | ND |  |

ND: Non-detectable.

Source: Chemical and Microbiological Laboratory, Industrial Technology Institute (ITI), Colombo 7, Sri Lanka.

Supplementary Table 3: A brief study assessment for each visit

|  | **Palm oil feeding period** | | | **Coconut oil feeding period** | | |
| --- | --- | --- | --- | --- | --- | --- |
|  | 0 week | 4^th^ week | 8^th^ week | 0 week | 4^th^ week | 8^th^ week |
| Informed consent form | √ | -- | -- | -- | -- | -- |
| Demographic data | √ | -- | -- | -- | -- | -- |
| Medical history taking | √ | -- | -- | -- | -- | -- |
| Height | √ | -- | -- | -- | -- | -- |
| Weight | √ | -- | √ | √ | -- | √ |
| Waist circumference | √ | -- | √ | √ | -- | √ |
| Hip circumference | √ | -- | √ | √ | -- | √ |
| 24-hour dietary recall | √ | -- | √ | √ | -- | √ |
| FFQ* | -- | √ | -- | -- | √ | √ |
| Physical Activity** | -- | √ | -- | -- | √ | -- |

*Data were taken from Food Frequency Questionnaire (FFQ)

**Data were taken from International Physical Activity Questionnaire (IPAQ)

Supplementary Table 4: Socio-demographic characteristics of the study population

| Variables |  | Total (*N*=37) | |
| --- | --- | --- | --- |
|  |  | *n* | % |
| Age | 18-30 years | 10 | 27.0 |
|  | 31-40 years | 12 | 32.4 |
|  | 41-50 years | 06 | 16.2 |
|  | 51-60 years | 07 | 18.9 |
|  | >60 years | 02 | 5.4 |
| Gender | Male | 14 | 37.8 |
|  | Female | 23 | 62.2 |
| Marital status | Married | 30 | 81.1 |
|  | Unmarried | 07 | 18.9 |
| BMI categories* (kgm^-2^) | < 18.5 (underweight) | 06 | 16.2 |
|  | 18.5- 24.9 (normal weight) | 20 | 54.1 |
|  | 25.0- 29.9 (overweight) | 07 | 18.9 |
|  | ≥ 30.0 (obese) | 04 | 10.8 |
| Number of family members | 1-3 | 12 | 32.4 |
|  | 4- 6 | 25 | 67.6 |
| Education level | Primary education | 03 | 8.1 |
|  | Secondary education | 07 | 18.9 |
|  | Tertiary education | 24 | 64.9 |
|  | Diploma/degree | 03 | 8.1 |
| Employment status | Employed | 11 | 29.7 |
|  | Self-employed | 16 | 43.2 |
|  | Unemployed | 10 | 27.1 |
| Smoking status | Former | 0 | 0 |
|  | Current | 0 | 0 |
| Alcohol consumption | Never | 32 | 0 |
|  | 1-2 times per month | 5 | 13.5 |
|  | 2-4 times per month | 0 | 0 |
| Monthly family income (in LKR) | < 25,000 | 05 | 13.5 |
|  | 25,000-49,999 | 14 | 37.8 |
|  | 50,000-99,999 | 14 | 37.8 |
|  | >100,000 | 04 | 10.8 |

Abbreviation: BMI- Body Mass Index; LKR- Sri Lankan Rupees

*BMI categories are based on World Health Organization classification

Supplementary Table 5: Family history of co-morbidities

| Family history of co-morbidities | | Response | Total (*N*=37) | |
| --- | --- | --- | --- | --- |
|  |  |  | *n* | % |
| Father | Dyslipidemia | Yes | 11 | 29.7 |
|  |  | No | 26 | 70.3 |
|  | Ischemic heart disease | Yes | 02 | 5.4 |
|  |  | No | 35 | 94.6 |
| Mother | Dyslipidemia | Yes | 07 | 18.9 |
|  |  | No | 30 | 81.1 |
|  | Ischemic heart disease | Yes | 02 | 5.4 |
|  |  | No | 35 | 94.6 |
| Siblings | Dyslipidemia | Yes | 01 | 2.7 |
|  |  | No | 36 | 97.3 |
|  |  | Don’t know | 09 | 24.3 |
|  | Ischemic heart disease | Yes | 01 | 2.7 |
|  |  | No | 30 | 81.1 |
|  |  | Don’t know | 06 | 16.2 |

**Supplementary content 1:**

**Estimation of oil dosage based on the calorie intake**

As an example; for a 2000 kcal diet, an average of 22 g of treatment oil per day was recommended to give 10% of energy from test oil. Therefore, for the individual who requires 2000 kcal of daily energy, nearly 24 mL (considering the specific gravity of coconut oil as 0.92) of test oil per day was regarded as the average amount of oil to be consumed by that person to obtain the required impact in the outcomes of variables.

**Supplementary content 2:**

**Oil compliance**:

**Monitoring the compliance of the oil intake**:

Also, adherence to the intervention plan by the subject was ensured through monitoring the volume remaining in the oil containers in order to examine any deviation between oil recorded, used, and supplied, checking oil recording sheet to ensure whether they completed the assigned oil amount within the day, conducting food recalls and regular interviews (including in-depth discussion if required) to tackle whether they used the assigned oil volume on daily basis, telephone communication to encourage participants to adhere to test oil volume and usual food habits daily. This allowed the investigators to review their compliance with oil intake and identify subjects who should be withdrawn from the study or removed from the final data analysis.

1. Compliance for number of days with treatment oil,

Compliance % (as number of days consumed) = [(no. of days with treatment oil – no. of days without treatment oil)/ No of days with treatment oil] x 100%

2) Compliance with the percentage of daily energy from test oil,

Compliance % (as a percentage of energy) = [Average daily energy intake from test oil (%) / Minimum percentage of recommended energy from test oil (which is equal to 10%)] x 100%

- Average daily energy intake from test oil (%) = [Average daily energy intake from test oil (kcal) / Average total daily energy intake from complete diet* (kcal)] x100%
- Average daily energy intake from test oil (kcal) = {Total volume of oil consumed for treatment period (mL)/ [(no. of days x number of members consumed per day)]} x 0.92** x (9 kcal/g)

*To calculate the average total daily energy intake from a complete diet, the mean of the 24-hour dietary recall which was taken 2 times in each treatment period as well as the energy from FFQ were considered separately.

** Specific gravity of coconut oil= 0.919; Specific gravity of palm oil= 0.916.

(As 10% of energy from test oil is the ‘minimum dosage’ that subject should consume per day, equal to or more than 10% of energy from test oil was considered as 100% compliance).

**Supplementary content 3:**

***Socio-demographic details:*** An investigator-administered questionnaire was used to collect the socio-demographic information of the participants which included age, gender, level of education, occupation, monthly family income, and other relevant information about the family, past medical history, and family medical history.

***Anthropometric assessment:*** All anthropometric measures (height, weight, WC, and HC) were taken with standard calibrated equipment and in accordance with recommended protocols (Center for  Disease  Control  and  Prevention, 2007) at the beginning of the first and second feeding periods and end of the first and second feeding periods, except height which was measured only at the beginning. These were all performed by a single trained investigator to minimise inter-observer variation.

*Height:* Height was measured to the closest 0.1cm as the greatest distance from heels to the topmost point on the head, with the participant standing barefoot and in the full inspiration, using a calibrated stadiometer (SECA 217 by SECA GmbH & Co. Kg. Hamburg, Germany).

*Bodyweight:* Body weight was measured to the nearest 0.1kg using a calibrated digital weighing scale (SECA cat no. 813 by SECA GmbH & Co. KG, Hamburg, Germany) with the participants wearing indoor light clothing. Two measurements were taken for each person and the average weight was considered the actual weight for each person. BMI was computed by dividing weight (in kilograms) by height squared (in meters).

*Body Mass Index (BMI):* BMI was calculated by the standard formula (weight (Kg) / Height^2^ (m^2^)). BMI categories are based on WHO classification (WHO, 2010)

*Waist circumference (WC):* WC was measured to the closest 0.1cm at the end of normal expiration using a non-elastic plastic flexible tape (SECA 203 by SECA GmbH & Co. Kg. Hamburg, Germany) midway between the iliac crest and the lower rib margin.

#### Hip circumference (HC): HC was measured to the closest 0.1cm using a plastic flexible tape (SECA 203 by SECA GmbH & Co. Kg. Hamburg, Germany) at the broadest level across the larger inter-trochanteric level.

#### Wait to hip ratio (WHR): WHR was calculated by standard equation: (WC (cm) / HC (cm)).
